# What are the appropriate methodological standards for international comparisons of health data?

**DOI:** 10.1186/s13584-017-0199-3

**Published:** 2018-01-08

**Authors:** Peter S. Hussey, Maria O. Edelen

**Affiliations:** RAND, 20 Park Plaza, Suite 920, Boston, MA 02540 USA

## Abstract

International comparisons of health systems are frequently used to inform national health policy debates. These comparisons can be used to gauge areas of strength and weakness in a health system, and to find potential solutions from abroad that can be applied locally. But such comparisons are methodologically fraught and, if not carefully performed and used, can be misleading.

In a recent IJHPR article, Baruch Levi has raised concerns about the use of international comparisons of self-reported health data in health policy debates in Israel. Self-reported health is one of the most robust and frequently used measures of health, and the OECD uses a commonly accepted measure specification, which has five response categories. Israel’s survey question, unlike the OECD measure specification, includes only four response categories. While this may be a valid method when applied over time as a scale within Israel, it creates problems for international comparison.

To improve comparability, Israel’s Central Bureau of Statistics could revise the survey question. However, revising the question would introduce a “break” in the data series that interrupts comparisons within Israel over time. Israeli policymakers therefore face a decision about priorities: is it more important to them to be able to track health status within Israel over time, or to be able to make meaningful comparisons to other countries? If the priority were international comparisons and the Israel survey was revised, a small study could be conducted among a sample of Israeli respondents to enable crosswalking of self-reported health responses from the four-point scale to the five-point scale. If the Central Bureau of Statistics does not revise its survey, the OECD should examine whether a stronger caveat is possible for its comparisons.

International comparisons of health systems are frequently used to inform national health policy debates. These comparisons can be used to gauge areas of strength and weakness in a health system, and to find potential solutions from abroad that can be applied locally. But such comparisons are methodologically fraught and, if not carefully performed and used, can be misleading. What standards should be applied so that international comparisons of health systems data are constructive, not misleading?

Baruch Levi highlights these issues in an examination of the use of international comparisons of self-reported health data in health policy debates in Israel [1]. The Organization for Economic Cooperation and Development (OECD), a leading source of international health systems data, publishes comparisons of self-reported perceived health based on surveys performed in multiple countries. This measure is also part of a composite measure calculated by the OECD, the “Better Life Index.”

The first standard that international comparisons should meet is the use of important, scientifically sound measures. This standard is met in this case: self-reported health is one of the most robust and frequently used measures of health, particularly in applications that call for a brief but broad assessment of general health, and has been shown to be correlated with other health outcomes [2].

A second standard is the use of an appropriate, rigorous measure specification. The OECD’s specification is consistent with the recommendation of groups including the World Health Organization and EURO-REVES 2 – but country-specific surveys can vary from this definition [3]. The OECD’s definition of the question uses a five-category Likert scale of responses: very good, good, fair, bad, or very bad. The OECD then calculates rates of positive responses (very good or good), neutral responses (fair), and negative responses (bad or very bad).

A third standard is whether data are collected consistently in a way that meets the specification. The OECD faces a considerable challenge in attempting to harmonize and compare health statistics from multiple countries. Even measures that use internationally recognized units of measurement, such as health spending, are extremely challenging to harmonize across countries: different currencies are used, with fluctuating exchange rates; different accounting methods are sometimes applied; and categories of spending are defined differently between countries. While international organizations can encourage harmonization across countries, such as the World Health Organization’s System of Health Accounts [4], it can be difficult for countries to make the investment needed for a significant change in health accounting systems.

Measures of self-reported health perhaps face even more significant harmonization challenges. Surveys responses can be affected by translation or cultural norms. Levi reports that the OECD self-reported health measure is based on questions from multiple surveys. These surveys use different wording for questions and responses and different response categories, making harmonization of the results for reporting difficult.

Israel’s survey question includes only four response categories, unlike the OECD measure specification, which uses five. While the survey question may be valid when applied over time within Israel, it creates problems for international comparison. Evidence is mixed on the extent to which a four-point scale without a neutral option produces different response distributions than a five-point scale with a neutral option [5], but it is likely that comparing rates of the top two responses between surveys with 4-point and 5-point scales would introduce bias. Without a neutral response option, respondents are forced to answer positively or negatively – and indeed, both positive and negative perceived health rates for Israel are high relative to OECD peers. On other measures of health status compared by the OECD, such as life expectancy, Israel is often close to the OECD average [6].

Given these differences, the question is how, or whether, to report the data. This is an instance of a longstanding debate over transparency in health care [7]. Advocates of transparency argue that since we will never achieve methodological perfection, it is better to start by airing the information we have. This will spur improvement in the methods that would never occur without publication [8]. Caveats can be added to note methodological issues that could bias comparisons.

Critics of transparency worry that the harms of publishing biased information will outweigh the benefits and, by destroying trust in health statistics, harm the longer-term enterprise of improving health care. Levi seems to take this view by stating that the “lack of methodological uniformity does not allow viable international comparison.” Once data are published, the publisher loses control over how they are used. Levi notes that in Israel, the media and government frequently cite the positive self-reported health statistics without including the caveats the OECD attaches to the data.

The standard for publication should be whether the potential harms outweigh the potential benefits – similar to judgments about the appropriateness of medical services [9]. In this case, the concerns highlighted by Levi are certainly significant, and it’s possible (but uncertain) that publication is on the wrong side of the benefit/harm equation.

To improve comparability, Israel’s Central Bureau of Statistics could revise the survey question. However, revising the question would introduce a “break” in the data series that interrupts comparisons within Israel over time. Israeli policymakers therefore face a decision about priorities: is it more important to them to be able to track health status within Israel over time, or to be able to make meaningful comparisons to other countries?

In some cases, it may be possible to use analyses to improve comparability of different measures, but that would likely be challenging here. A small study could be conducted among a sample of Israeli respondents to enable crosswalking of self-reported health responses from the four-point scale to the five-point scale. This could help to understand the extent of a series break. However, it would likely be impossible to use such a study to map responses on a four-point scale to the OECD categories of positive and negative responses calculated from five-point scales with neutral options. Thus, this analytic repair might smooth out the transition, but would not be able to resolve the biases of the four-point scale discussed by Levi.

Short of removing the data, the OECD should examine whether a stronger caveat is possible. This might include removing the self-reported health measure from Israel’s Better Life Index (i.e., not reporting the composite index for Israel), since it could obscure the issues with the underlying data. The OECD could consider adding or changing the categories of health status it reports; for example, responses could be reported for each response separately, or as a continuous measure.

## Conclusions

Comparative information from other OECD countries could be useful in Israel health policy planning. In order to make more meaningful comparisons, however, Israel will need to make changes to its long-running health survey to conform with international norms for surveys of self-reported health status.

## References

[1] Levi B (2017). Perceived health status in a comparative perspective: methodological limitations and policy implications for Israel. Isr J Health Policy Res.

[2] Au N, Johnston DW (2014). Self-assessed health: what does it mean and what does it hide?. Soc Sci Med.

[3] Jylhä M (2009). What is self-rated health and why does it predict mortality? Towards a unified conceptual model. Soc Sci Med.

[4] World health organization. Health Accounts. Available at: http://www.who.int/health-accounts/en/. Last accessed 16 Nov 2017.

[5] Leung S-O (2011). A comparison of psychometric properties and normality in 4-, 5-, 6-, and 11-point Likert scales. J Soc Serv Res.

[6] Organization for Economic Cooperation and Development (2017). Health at a glance 2017.

[7] Marshall MN, Shekelle PG, Leatherman S, Brook RH (2000). The public release of performance data - what do we expect to gain? A review of the evidence. J Am Med Assoc.

[8] Marcotte BJ, Fildes AG, Thompson M, Binder L. U.S. health care reform can’t wait for quality measures to be perfect. Harvard Business Review. Available at: https://hbr.org/2017/10/u-s-health-care-reform-cant-wait-for-quality-measures-to-be-perfect#. Last accessed 16 Nov 2017.

[9] Brook RH, Chassin MR, Fink A, Solomon DH, Kosecoff J, Park R (1986). A method for the detailed assessment of the appropriateness of medical technologies. Int J Technol Assess Health Care.

